# New tumor regression grade for rectal cancer after neoadjuvant therapy and radical surgery

**DOI:** 10.18632/oncotarget.6008

**Published:** 2015-10-19

**Authors:** Jun Li, Hao Liu, Junjie Hu, Sai Liu, Jie Yin, Feng Du, Jiatian Yuan, Bo Lv

**Affiliations:** ^1^ General Surgery Department, Affiliated Hospital/Clinical Medical College of Chengdu University, Chengdu, People's Republic of China; ^2^ General Surgery Department, 2nd Affiliated Hospital of Jilin University, Changchun, People's Republic of China; ^3^ Gastrointestinal Tumor Surgery, Hubei Cancer Hospital, Wuhan, People's Republic of China; ^4^ Surgical Department of Gastrointestinal Diseases, Beijing Youan Hospital, Capital Medical University, Beijing, People's Republic of China; ^5^ General Surgery Department, Xuzhou Central Hospital, Xuzhou, People's Republic of China; ^6^ Internal Medicine-Oncology, Cancer Institute/Hospital, Peking Union Medical College and Chinese Academy of Medical Sciences, Beijing, People's Republic of China

**Keywords:** tumor regression grading, rectal cancer, neoadjuvant therapy

## Abstract

In this retrospective study, we defined a new tumor regression grade (NTRG), which we used to evaluate the prognosis of patients with locally advanced rectal cancer who received neoadjuvant therapy and then underwent radical surgery between June 2004 and October 2011. Calculated as the TRG plus a lymph node score, the NTRG was determined for 347 patients: NTRG 0, 46 patients (13.3%); NTRG 1, 63 (18.2%); NTRG 2, 183 (52.7%); NTRG 3, 30 (8.6%); NTRG 4, 25 (7.2%). Among this group, 45 (97.8%) NTRG 0, 56 (88.9%) NTRG 1, 148 (80.9%) NTRG 2, 24 (66.7%) NTRG 3, and 10 (40.0%) NTRG 4 patients experienced 5-year disease-free survival. We also found that NTRG is significantly associated with 5-year local recurrence, distant metastasis and disease-free survival (*P* = 0.004, 0.007 and 0.039, respectively). The NTRG may thus be an independent prognostic factor for oncologic outcomes in rectal cancer patients after neoadjuvant therapy and radical surgery, but this conclusion must be validated in randomized trials.

## INTRODUCTION

Rectal cancer patients receiving neoadjuvant therapy (preoperative chemoradiotherapy or radiotherapy only) may experience late local recurrence and distant metastasis [1]. Long-term follow-up of participants in the CAO/ARO/AIO-94 Rectal Cancer Trial revealed a continuous increase in local recurrence for up to 10 years [2]. There is thus substantial interest in short-term surrogate end points. A large number of neoadjuvant chemoradiotherapy trials have explored the use of the pathologic complete response (pCR) and/or tumor regression grade (TRG) as primary end points, and various grading systems have been proposed. The TRG is based on pathologic evaluation of specimens obtained during surgery, but the grade is not based any standard definitions, which makes it challenging to interpret. In fact, one recent study reported that there is poor agreement among the different TRG systems used [3]. Moreover, the TRG does not account for lymph node involvement, which is an important determinant of prognosis [1]. We therefore attempted to develop a new method for evaluating oncological outcomes that takes into consideration both the TRG and lymph node status.

## RESULTS

### Patient characteristics and association of NTRG with clinicopathologic factors

A total of 347 patients with stage II or III rectal cancer who received radical surgery within 7.5 ± 0.2 weeks (range: 6–8 weeks) after neoadjuvant therapy were identified in this retrospective study. With this group, each patient was assigned a new tumor regression grade (NTRG) as follows: NTRG 0, 46 patients [13.3%]; NTRG 1, 63 patients [18.2%]; NTRG 2, 183 patients [52.7%]; NTRG 3, 30 patients [8.6%]; NTRG 4, 25 patients [7.2%]). cT stage was significantly predictive of NTRG (*P* = 0.003) (Table 1). A pCR of the primary tumor (NTRG 0) was seen in 21.8% of patients with cT2 disease, 12.3% of those with cT3, and 1.2% of those with cT4.

**Table 1:** New tumor regression grade (NTRG) and patients after neoadjuvant therapy and radical surgery

NTRG	Patients No. (%)	TRG + LN Score		Local Recurrence	Distant Metastasis	All Failure
		Score	Patients No. (%)	No. (%)	No. (%)	No. (%)
0	46 (13.3%)	0+0	46 (100%)	1 (2.2%)	0 (0%)	1 (2.2%)
1	63 (18.2%)	0+1	13 (20.6%)	1 (7.7%)	1 (7.7%)	7 (11.1%)
		1+0	50 (79.4%)	1 (2.0%)	4 (8.0%)	
2	183 (52.7%)	0+2	10 (5.5%)	2 (20.0%)	1 (10.0%)	35 (19.1%)
		2+0	102 (55.7%)	7 (6.7%)	11 (10.8%)	
		1+1	71 (38.8%)	10 (14.3%)	4 (5.6%)	
3	30 (8.6%)	1+2	12 (40.0%)	2 (16.7%)	2 (16.7%)	10 (33.3%)
		2+1	18 (60.0%)	2 (11.1%)	4 (22.2%)	
4	25 (7.2%)	2+2	25 (100%)	8 (32.0%)	7 (28.0%)	15 (60.0%)

Note: The rates of local recurrence and distant metastasis in the NTRG 1–3 subgroups were similar (all *P* > 0.05). We therefore considered the NTRG 1 (0+1; 1+0), 2 (0+2; 2+0; 1+1) and 3 (1+2; 2+1) to be a whole, respectively.

The association of the NTRG with histopathologic factors is summarized in Table 2. Radical resection of the primary tumor (R0) was performed in 100% of patients. The NTRG was significantly related to ypT stage, ypN stage, lymphatic or venous invasion, and tumor deposits (*P* < 0.001 for all). No significant association was found between the NTRG and the degree of tumor differentiation after radical surgery. Given that pCR means there is no lymphatic invasion, venous invasion or tumor deposits, statistical analysis was performed with NTRG 1–4. And considering that NTRG 0 (pCR) corresponds to ypT0, ypT 4, ypN0 and ypN2, additional statistical analysis was restricted to NTRG 1–3 for those variables.

**Table 2:** Association of NTRG with pretreatment factors and tumor characteristics

Variable	NTRG 0		NTRG 1		NTRG 2		NTRG 3		NTRG 4		Total	*P*
	No.	%	No.	%	No.	%	No.	%	No.	%	No.	
Overall	46	13.3	63	18.2	183	52.7	30	8.6	25	7.2	347	
Age, years												
≤60	26	13.6	33	17.4	99	52.1	18	9.5	14	7.4	190	0.965
>60	20	12.7	30	19.1	84	53.5	12	7.6	11	7.0	157	
Gender												
Male	30	14.5	41	19.8	105	50.7	16	7.7	15	7.2	207	0.689
Female	16	11.4	22	15.7	78	55.7	14	10.0	10	7.1	140	
Distance from anal verge, cm												
≤5	26	13.7	35	18.5	96	50.8	14	7.4	18	9.5	189	0.374
5–10	20	12.7	28	17.7	87	55.1	16	10.1	7	4.4	158	
Preoperative CEA												
<5 ng/ml	27	14.2	30	15.8	106	55.8	16	8.4	11	5.8	190	0.331
≥5 ng/ml	17	12.5	28	20.6	70	51.5	11	8.1	10	7.4	136	
unknown	2	9.5	5	23.8	7	33.3	3	14.3	4	19.0	21	
Preoperative NT												
Chemoradiotherapy	31	14.6	40	18.8	110	51.6	17	8.0	15	7.0	213	0.871
Radiotherapy only	15	11.2	23	17.2	73	54.5	13	9.7	10	7.5	134	
cT stage												
cT2	30	21.8	30	21.8	67	48.5	7	5.1	4	2.9	138	0.003
cT3	15	12.3	23	18.9	70	57.4	7	5.7	7	5.7	122	
cT4	1	1.2	10	11.9	33	39.3	16	19.0	24	28.6	84	
unknown	0		0		3	100.0	0		0		3	
cN stage												
cN0	25	14.2	36	20.6	92	52.6	11	6.3	11	6.3	175	0.392
cN+	21	12.2	27	15.6	91	52.9	19	11.0	14	8.1	172	

### NTRG as a prognostic factor for DFS

**Figure 1:**
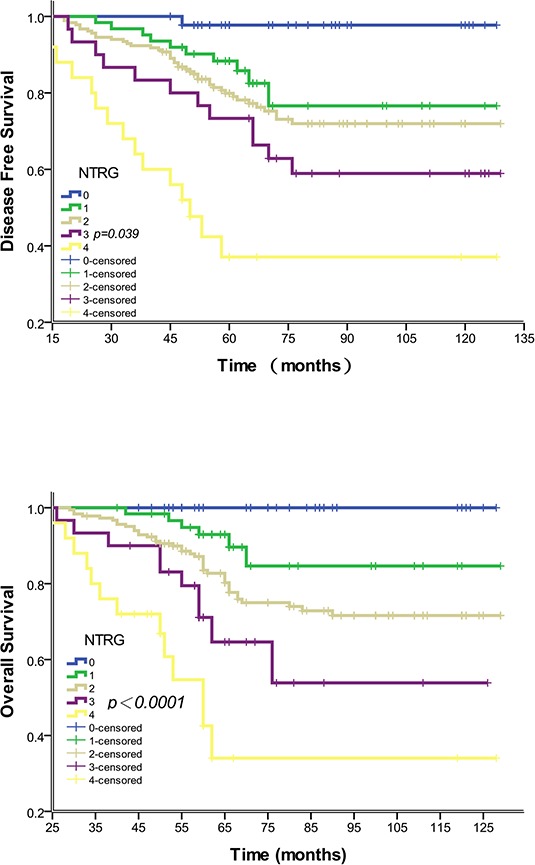
Association of NTRG with disease-free and overall survival **A.** Disease-free survival curves showing a significant relation to NTRG. Data for all 347 cases were available. The 5-year disease-free survival rates for NTRG 0–4 were 97.8% (45/46), 88.9% (56/63), 80.9% (148/183), 66.7% (20/30) and 40.0% (10/25), respectively (*P* = 0.039). NTRG 1 and 2 had similar 5-year disease-free survival rates (*P* > 0.05). **B.** Overall survival curves showing a significant relation to NTRG. The 5-year overall survival rates for NTRG 0–4 were 100.0%, 82.6%, 73.4% 55.4% and 24.2%, respectively (*P* < 0.0001). NTRG 1 and 2 had similar 5-year overall survival rates (*P* > 0.05).

The 5-year DFS for the 347 patients was 80.4% after radical surgery. Local recurrence was detected in 34 patients, and distant metastasis was detected in another 34 patients. No patients suffered both local recurrence and distant metastasis. Forty-five (97.8%) patients with NTRG 0, 56 (88.9%) with NTRG 1, 148 (80.9%) with NTRG 2, 24 (66.7%) with NTRG 3 and 10 (40.0%) with NTRG 4 experienced a 5-year DFS. TRG was significantly associated with 5-year distant metastasis (*P* = 0.035), but not 5-year local recurrence or DFS rates (*P* = 0.531, 0.576, respectively). By contrast, the NTRG was significantly associated with 5-year local recurrence, 5-year distant metastasis and 5-year DFS (*P* = 0.004, 0.007 and 0.039, respectively). Univariate analysis showed that the ypT and ypN stages, lymphatic invasion and venous invasion all correlated significantly with DFS (all *P* < 0.05) (Table 3). In a multivariate analysis of all significant factors from the univariable analysis, except ypN and TRG, both ypT and NTRG were found to be independent risk factors of 5-year local recurrence, distant metastasis and DFS (Table 4). Disease-free and overall survival curves for NTRG are shown in Figure 1A and 1B.

**Table 3:** Association of NTRG with pathological factors after neoadjuvant therapy and radical surgery

Variable	NTRG 0		NTRG 1		NTRG 2		NTRG 3		NTRG 4		Total	*P*
	No.	%	No.	%	No.	%	No.	%	No.	%		
Overall	46	13.3	63	18.2	183	52.7	30	8.6	25	7.2	347	
ypT stage												
ypT0	46	100.0	13	20.6	10	5.5	0	0	0	0	69	<0.001^a^
ypT1	0	NA	25	39.7	96	52.5	0	0	0	0	121	
ypT2	0	NA	15	23.8	60	32.8	20	66.7	0	0	95	
ypT3	0	NA	7	11.1	15	8.2	10	33.3	0	0	32	
ypT4	0	NA	3	4.8	2	1.1	0	0	25	100.0	30	
ypN stage												
ypN0	46	100.0	50	79.4	102	55.7	0	0	0	0	198	<0.001^a^
ypN1	0	NA	13	20.6	71	38.8	18	60.0	0	0	102
ypN2	0	NA	0	0	10	5.5	12	40.0	25	100.0	47	
Tumor differentiation degree												
poor	7	15.2	17	27.0	40	21.9	7	23.3	9	36.0	80	0.869
moderate	18	39.1	20	31.7	61	33.3	14	46.7	7	28.0	120	
well	21	45.7	26	41.3	82	44.8	9	30	9	36.0	147	
Lymphatic invasion												
negative	46	100.0	50	79.4	145	79.2	17	56.7	16	64.0	274	<0.001^b^
positive	0	NA	13	20.6	38	20.8	13	43.3	9	36.0	73	
Venous invasion												
negative	46	100.0	46	73.0	123	67.2	17	56.7	11	44.0	243	<0.001^b^
positive	0	NA	17	27.0	60	32.8	13	43.3	14	56.0	104	
Tumor deposits												
negative	46	100.0	50	79.4	151	82.5	20	66.7	12	48.0	279	<0.001^b^
positive	0	NA	13	20.6	32	17.5	10	33.3	13	52.0	68	

Abbreviation: NA, not applicable.

a Statistical analysis was restricted to NTRG 1–3 for these variables.

b Statistical analysis was restricted to NTRG 1–4 for these variables.

**Table 4:** Influence of different clinical and pathologic factors on 5-year prognosis after neoadjuvant therapy and radical surgery

Variables	No. of Patients	LRNo. (%)	5-Year LR free rate	*P*	DMNo. (%)	5-Year DM free rate	*P*	All Failure No. (%)	5-Year DFS rate	*P*
Overall	347	34(9.8%)	91.2%		34(9.8%%)	91.2%		68(19.6%)	80.4%	
Age, years										
≤60	190	19(10.0%)	90.0%	0.900	18(9.5%)	90.5%	0.077	37(19.5%)	80.5%	0.983
>60	157	15(9.6%)	90.4%		16(10.2%)	89.8%		31(19.7%)	80.3%	
Gender										
Male	207	23(11.1%)	88.1%	0.363	22(10.6%)	89.4%	0.566	45(21.7%)	78.3%	0.688
Female	140	11(7.9%)	92.1%		12(8.6%)	91.4%		23(26.4%)	83.6%	
ypT stage										
ypT0	69	1(1.4%)	98.6%	0.014	1(1.4%)	98.6%	0.001	2(2.9%)	97.1%	0.024
ypT1	121	9(7.4%)	92.6%		9(7.4%)	92.6%		18(14.9%)	85.1%	
ypT2	95	11(11.6%)	88.4%		8(9.5%)	90.5%		19(20.0%)	80.0%	
ypT3	32	6(18.8%)	81.2%		6(18.8%)	81.2%		12(37.5%)	62.5%	
ypT4	30	7(23.3%)	76.7%		10(33.3%)	66.7%		17(56.7%)	43.3%	
ypN stage										
ypN0	198	9(4.5%)	95.5%	0.001	15(7.6%)	92.4%	0.044	24(12.1%)	87.9%	<0.0001
ypN1	102	13(12.7%)	87.3%		9(8.8%)	91.2%		12(21.6%)	78.4%	
ypN2	47	12(25.5%)	74.5%		10(21.3%)	78.7%		22(46.8%)	53.2%	
Tumor differentiation degree										
poor	80	13(16.3%)	83.7%	0.089	13(16.3%)	83.7%	0.089	26(32.5%)	67.5%	0.468
moderate	120	12(10.0%)	90.0%		12(10.0%)	90.0%		24(20.0%)	80.0%	
well	147	9(6.1%)	93.9%		9(6.1%)	93.9%		18(21.2%)	87.8%	
Lymphatic invasion										
negative	274	13(4.7%)	95.3%	<0.0001	13(4.7%)	95.3%	<0.0001	26(9.5%)	90.5%	0.001
positive	73	21(28.8%)	71.2%		21(28.8%)	71.2%		4257.5(%)	42.5%	
Venous invasion										
negative	243	9(3.7%)	92.3%	<0.0001	11(4.5%)	95.5%	<0.0001	20(8.2%)	91.8%	0.005
positive	104	25(24.0%)	76.0%		23(22.1%)	77.9%		48(46.2%)	53.8%	
Tumor deposits										
negative	279	26(9.3%)	80.7%	0.584	25(9.0%)	91.0%	0.341	51(11.1%)	88.9%	0.406
positive	68	8(11.8%)	88.2%		9(13.2%)	86.8%		17(25.0%)	75.0%	
Postoperative chemotherapy										
Yes	285	22(7.7%)	92.3%	0.014	25(8.8%)	91.2%	0.219	47(16.5%)	83.5%	0.287
No	62	12(19.4%)	80.6%		9(14.5%)	85.5%		21(33.9%)	66.1%	
TRG										
0(total)	69	4(5.8%)	94.2%	0.531	2(2.9%)	97.1%	0.035	6(8.7%)	91.3%	0.576
1(intermediate)	133	13(9.8%)	90.2%		10(7.5%)	92.5%		23(17.3%)	82.7%	
2(minor and no)	155	17(11.0%)	89.0%		22(14.2%)	85.8%		29(26.5%)	73.5%	
NTRG										
0	46	1(2.2%)	97.8%	0.004	0(0%)	100.0%	0.007	1(2.2%)	97.8%	0.039
1	63	2(3.2%)	96.8%		5(7.9%)	92.1%		7(11.1%)	88.9%	
2	183	19(10.4%)	89.6%		16(8.7%)	91.3%		35(19.1%)	80.9%	
3	30	4(13.3%)	86.7%		6(20.0%)	80.0%		10(33.3%)	66.7%	
4	25	8(32.0%)	68.0%		7(28.0%)	72.0%		15(60.0%)	40.0%	

Abbreviations: LR, local recurrence; DM, distant metastasis.

**Figure 2:**
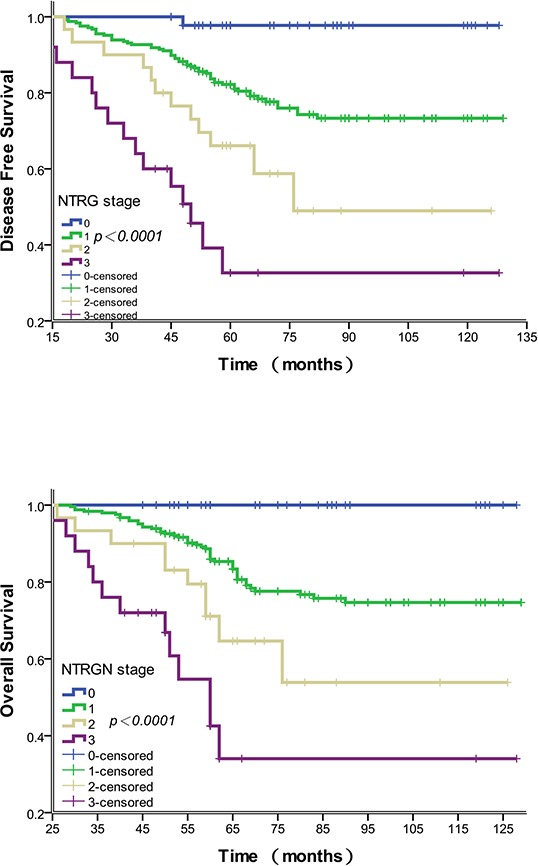
Association of the modified NTRG with disease-free and overall survival **A.** Disease-free survival curves showing a significant relation to the modified NTRG. The 5-year disease-free survival rates for modified NTRG 0–3 were 97.8%, 82.9%, 66.7% and 40.0%, respectively (*P* < 0.0001). **B.** Overall survival curves showing a significant relation to the modified NTRG. The 5-year overall survival rates for NTRG 0–3 were 100.0%, 75.5%, 55.4% and 24.2%, respectively (*P* < 0.0001).

When comparing the 5-year DFS rates among the five NTRG groups, we found that NTRG 1 and 2 gave similar results (88.9% vs. 80.9%, *P* = 0.146). We therefore combined the patients with NTRG 1 and 2 into a new group. The resultant modified NTRG system is shown in Table 5. The 5-year DFS rates for NTRG 0 vs. NTRG 1 were 97.8% vs. 82.9% (Χ^2^ = 45.965, *P* < 0.0001), NTRG 1 vs. NTRG 2 (Χ^2^ = 4.505, *P* = 0.034) and NTRG 2 vs. NTRG 3 (Χ^2^ = 3.911, *P* = 0.047). Disease-free and overall survival curves for the modified NTRGs are shown in Figure 2A and 2B.

**Table 5:** Multivariate analysis for three end points after NT and radical surgery

Variables	5-Year Local Recurrence			5-Year Distant Metastasis			5-Year Disease Free Survival		
	HR	95.0% CI	*P*	HR	95.0% CI	*P*	HR	95.0% CI	*P*
	0.42	(0.36 to 0.81)	0.022	0.51	(0.35 to 0.87)	0.038	0.57	(0.41 to 0.76)	0.033
Lymphatic invasion	1.51	(0.97 to 2.16)	0.084	1.12	(0.91 to 1.35)	0.482	1.22	(0.96 to 1.32)	0.069
Venous invasion	1.23	(0.85 to 1.52)	0.903	0.873	(0.63 to 1.05)	0.679	1.09	(0.92 to 1.25)	0.843
Postoperative chemotherapy	1.09	(0.94 to 1.37)	0.053		—			—	
NTRG	1.63	(1.32 to 1.99)	0.017	1.39	(1.14 to 1.82)	0.031	1.86	(1.51 to 2.24)	0.026

Note: The Cox model excluded both ypN and TRG, which were related to the NTRG. We also used another Cox model, which included the ypN and TRG, but not NTRG. That model showed that TRG was not an independent predictor for the three end points, but ypN was (not shown in the table).

**Table 6:** The modified NTRG system for rectal cancer after NCRT and explanation

Modified NTRG stage	Explanation	Patients No. (%)	Failure cases No. (%)	5-year Disease Free Survival
0 (score 0)	pCR (TRG0+ypN0)	46 (13.3%)	1 (2.2%)	97.8%
1 (score 1+2)	TRG0+ypN1/2; TRG1+ypN 0/1; TRG2+ypN0	246 (70.9%)	42 (17.1%)	82.9%
2 (score 3)	TRG1+ypN2;TRG2+ypN1	30 (8.6%)	10 (33.3%)	66.7%
3 (score 4)	TRG2+ypN2	25 (7.2%)	15 (60.0%)	40.0%

Note: The 5-year DFS rates for failure in the NTRG 0-3 were significantly different (*P* < 0.0001), and the 5-year DFS rates for NTRG 0 vs. NTRG 1 (*P* < 0.0001), NTRG 1 vs. NTRG 2 (*P* = 0.034) and NTRG 2 vs. NTRG 3 (*P* = 0.047) were with statistically significant differences.

## DISCUSSION

Tumor regression after neoadjuvant therapy for rectal cancer can vary considerably. Whereas some patients show a complete absence of tumor cells, others exhibit a mass of tumor cells with little or no regressive changes [4]. Tumor regression is reportedly associated with the specifics of the preoperative treatments, including the overall radiation dose, whether the radiation was combined with chemotherapy, and the time interval between the therapy and surgery [5, 6]. In our study, 61.4% (213/347) of patients received neoadjuvant chemoradiotherapy, while the rest receive radiation only. Notably, our analysis showed no significant difference in NTRG between the two preoperative treatment schedules (*P* = 0.871). Considering that overall radiation dose (50 Gy) and the interval between preoperative therapy and surgery (6–8 weeks) were similar for all patients in our study, this suggests differences in NTRG likely reflect the characteristics of the individual tumors.

We found that the TRG correlated with 5-year distant metastasis, but that it was unlikely that the TRG was a prognostic factor for 5-year local recurrence or DFS. Based on data from the CAO/ARO/AIO-94 trial, Rodel et al. [7] concluded in 2005 that the TRG could be a risk factor for 5-year distant metastasis and DFS, after which the study group [2] updated the results, approving the TRG as a significant prognostic factor for 10-year distant metastasis and DFS. Additionally, Mace AG et al. [8] declared that the American Joint Committee on Caner/College of American Pathologists grade remains an independent predictor of overall survival, DFS and cumulative all failure (all *P* < 0.001). Our findings, however, indicate that TRG may not be significantly associated with 5-year local recurrence or DFS. These differences may reflect an inadequate number of patients in the present study, the different regression scoring systems used and/or differences in the treatment regimens, including the radiation dose, medications used in chemotherapy and pathology practices, as well as differences in the duration between neoadjuvant therapy and surgery. In fact, Kalady et al. [9] reported that patients with an incomplete response at 6 weeks may show a pCR at 12 weeks. Accordingly, the time interval between neoadjuvant therapy and radical surgery was a key determinant of pCR, which could impact TRG classification and interfere with the trial results. For those reasons, we think that, by itself, the TRG is not a stable prognostic factor.

The widespread implementation of a number of grading systems has been hindered by a lack of standardization. In addition, interpretation of the TRG assigned by a pathologist may be challenging due to the subjective nature of the histological interpretation of the response, which could potentially lead to unreproducible findings. For example, one recent study reported poor agreement among experienced pathologists, irrespective of the TRG system used [3]. What's more, most TRG systems fail to independently correlate with oncological outcomes [10, 11]. Perhaps we need a new TRG system with which to evaluate the prognosis of rectal cancer patients after neoadjuvant therapy and radical resection. Unlike the TRG, the NTRG takes into consideration both the primary tumor within the rectal wall and regional lymph nodes. Our data indicate that the NTRG is a significant risk factor for 5-year local recurrence, distant metastasis and DFS, as well as an independent factor affecting those three end points.

However, reproducibility is a key issue with any medical test or procedure, and the utility of the NTRG remains unclear. The results from this study are constrained by all the inherent flaws of retrospective research, some of which could lead to bias. These include the limited number of patients enrolled and differences in the neoadjuvant therapy regimens and postoperative chemotherapy. The ideal trial design to assess the NTRG system would be a prospective and randomized clinical trial. Nevertheless, we believe our outcome data for the NTRG are encouraging, and our method may provide a new way to assess likely oncologic outcomes in rectal cancer patients after neoadjuvant therapy and radical surgery. We have therefore initiated a randomized clinical trial to carefully evaluate the validity of our findings. All of the trial participants have advanced and resectable rectal cancer, and we are using the same preoperative chemoradiotherapy and postoperative chemotherapy for all patients. But for those enrolled into Group A there is a 12-week interval between completion of the preoperative therapy and surgery, while the interval is 8 weeks for those in Group B. Our purpose is to assess the validity of our earlier conclusions, determine whether the NTRG is superior to the existing TRG systems, and assess and compare the accuracy of the NTRG as a predictor of rectal cancer patient outcome at two intervals between completion of neoadjuvant therapy and radical surgery.

In sum, the TRG system is a prognostic factor for distant metastasis in rectal cancer after neoadjuvant therapy and radical resection, but it failed to predict the risk of local recurrence and distant metastasis in our study. Our results suggest the NTRG may be an independent risk factor predictive of oncological outcome in rectal cancer patients after neoadjuvant therapy and radical surgery, but the validity and reproducibility of this result must be tested in randomized trials.

## MATERIALS AND METHODS

This study was not considered to constitute an additional risk for enrolled patients. Approval was obtained from the appropriate ethics committees at all the participating study centers before the study started.

### Patients

We examined the records of 347 patients with primary mid-rectal or distal rectal cancer who had received preoperative neoadjuvant therapy followed by radical surgery at four study sites between June 2004 and October 2011. The study inclusion/exclusion criteria were: (1) rectal adenocarcinoma confirmed by surgical resection with a total mesorectal excision; (2) locally advanced resectable disease (clinical stage II or III) with an inferior tumor margin located no farther than 10 cm from the anal verge; (3) no evidence of distant metastasis; and (4) patients were administered neoadjuvant therapy.

### Neoadjuvant and adjuvant therapies

There is currently no international consensus with regard to the indications for neoadjuvant chemoradiation therapy. Therefore, patients managed with preoperative radiochemotherapy or preoperative radiotherapy alone were identified in our retrospective study. All patients received preoperative radiotherapy (50 Gy/2 Gy/25 f). Among those, 213 (61.4%) patients were concurrently treated with chemotherapy (capecitabine, 825 mg/m^2^/bid), and the rest received radiotherapy alone. All patients received the same capecitabine regimen (1000 mg/m^2^/bid, d1–14, 4–6 cycles) 3 weeks after radical surgery, except 62 (17.9%) who rejected chemotherapy due to their older age, poor physical condition or side effects.

### Pathologic examination

All pathological sections from resected specimens were examined by local pathologists from four hospitals who were blinded to the patients' clinical outcomes. The specimens were evaluated according to a standardized protocol that included 7^th^ AJCC TNM category, stage grouping, numbers of examined and involved lymph nodes, presence or absence of lymphatic or venous invasion, tumor deposits and TRG. A negative margin was scored as R0 resection, microscopic involvement of margins was scored R1, and gross residual tumor was scored as R2. After the reference pathologist evaluated the pathological sections, the scores were recorded using a standardized document.

### New tumor regression grade

Primary tumor regression was evaluated by determining the amount of viable tumor vs. fibrotic tissue in pathological sections. According to Dworak et al. [12], this can range from no tumor regression to a complete response with no viable tumor detected. The three TRGs were as follows: grade 0, total regression (no viable tumor cells; fibrotic mass only); grade 1, intermediate regression; grade 2, minor regression (dominant tumor mass with obvious fibrosis ≤25% of tumor mass) and no regression. In addition, lymph node status was classified as follows: score 0, no positive lymph nodes; score 1, 1–3 positive nodes; score 2, ≥4 positive nodes. The NTRG was calculated using the TRG classification plus the lymph node status score (Table 1).

### Follow-up

Follow-up results were collected from all four hospitals' databases. The end point of the follow-up was March 2015. The median duration of follow-up was 60 months (26–129 months).

### Statistical analysis

Statistical analysis was performed using SPSS software (version 18). Local recurrence and distant metastasis were analyzed for all eligible patients who received R0 resection and who were without detectable distant metastasis at the time of surgery after neoadjuvant therapy. All time-to-event end points were measured from the date of radical surgery. DFS was calculated from the time of radical resection to the discovery of evidence of recurrence and/or distant metastasis. Differences were evaluated using the log-rank test. Local recurrence and distant metastasis were analyzed as cumulative incidences. Mutivariable analysis was performed using the Cox proportional hazards model. All significant variables in the univariable analysis were included in the multivariable Cox regression models in a forward-step procedure. The variables were entered into the regression models in order according to their clinical relevance with increasing complexity, and significance was assessed using analysis of variance. A two-sided *P* value less than 0.05 was considered significant.

## References

[1] Sargent DJ, Patiyil S, Yothers G, Haller DG, Gray R, Benedetti J, Buyse M, Labianca R, Seitz JF, O'Callaghan CJ, Francini G, Grothey A, O'Connell M (2007). End points for colon cancer adjuvant trials: Observations and recommendations based on individual patient data from 20,898 patients enrolled onto randomized trials from the ACCENT Group. J Clin Oncol.

[2] Fokas E, Liersch T, Fietkau R, Hohenberger W, Beissbarth T, Hess C, Becker H, Ghadimi M, Mrak K, Merkel S, Raab HR, Sauer R, Wittekind C2 (2014). Tumor regression grading after preoperative chemoradiotherapy for locally advanced rectal carcinoma revisited: updated results of the CAO/ARO/AIO-94 trial. J Clin Oncol.

[3] Chetty R, Gill P, Govender D, Bateman A, Chang HJ, Deshpande V, Driman D, Gomez M, Greywoode G, Jaynes E, Lee CS, Locketz M, Rowsell C (2012). International study group on rectal cancer regression grading: interobserver variability with commonly used regression grading systems. Hum Pathol.

[4] Chetty R, Gill P, Bateman AC, Driman DK, Govender D, Bateman AR, Chua YJ, Greywoode G, Hemmings C, Imat I, Jaynes E, Lee CS, Locketz M (2012). Pathological grading of regression: An International Study Group perspective. J Clin Pathol.

[5] Moore HG, Gittleman AE, Minsky BD, Wong D, Paty PB, Weiser M, Temple L, Saltz L, Shia J, Guillem JG (2004). Rate of pathologic complete response with increased interval between preoperative combined modality therapy and rectal cancer resection. Dis Colon Rectum.

[6] Mohiuddin M1, Regine WF, John WJ, Hagihara PF, McGrath PC, Kenady DE, Marks G (2000). Preoperative chemoradiation in fixed distal rectal cancer: Dose time factors for pathological complete response. Int J Radiat Oncol Biol Phys.

[7] Rödel C, Martus P, Papadoupolos T, Füzesi L, Klimpfinger M, Fietkau R, Liersch T, Hohenberger W, Raab R, Sauer R, Wittekind C (2005). Prognostic significance of tumor regression after preoperative chemoradiotherapy for rectal cancer. J Clin Oncol.

[8] Mace AG, Pai RK, Stocchi L, Kalady MF (2015). American Joint Committee on Cancer and College of American Pathologists regression grade: a new prognostic factor in rectal cancer. Dis Colon Rectum.

[9] Kalady MF, de Campos-Lobato LF, Stocchi L, Geisler DP, Dietz D, Lavery IC, Fazio VW (2009). Predictive factors of pathologic complete response after neoadjuvant chemoradiation for rectal cancer. Ann Surg.

[10] Beddy D, Hyland JM, Winter DC, Lim C, White A, Moriarty M, Armstrong J, Fennelly D, Gibbons D, Sheahan K (2008). a simplified tumor regression grade correlates with survival in locally advanced rectal carcinoma treated with neoadjuvant chemoradiotherapy. Ann Surg Oncol.

[11] Abdul-Jalil KL, Sheehan KM, Kehoe J, Cummins R, O'Grady A, McNamara DA, Deasy J, Breathnach O, Grogan L, O'Neill BD, Faul C, Parker I, Kay EW (2014). the prognostic value of tumour regression grade following neoadjuvant chemoradiation therapy for rectal cancer. Colorectal Dis.

[12] Dworak O, Keilholz L, Hoffmann A (1997). Pathological features of rectal cancer after preoperative radiochemotherapy. Int J Colorectal Dis.

